# PhaeoEpiView: an epigenome browser of the newly assembled genome of the model diatom *Phaeodactylum tricornutum*

**DOI:** 10.1038/s41598-023-35403-1

**Published:** 2023-05-23

**Authors:** Yue Wu, Timothée Chaumier, Eric Manirakiza, Alaguraj Veluchamy, Leila Tirichine

**Affiliations:** 1 Nantes Université, CNRS, US2B, UMR 6286, 44000 Nantes, France; 2 St Jude Children’s Research Hospital, Memphis, TN USA

**Keywords:** Histone post-translational modifications, Marine biology

## Abstract

Recent advances in DNA sequencing technologies particularly long-read sequencing, greatly improved genomes assembly. However, this has created discrepancies between published annotations and epigenome tracks, which have not been updated to keep pace with the new assemblies. Here, we used the latest improved telomere-to-telomere assembly of the model pennate diatom *Phaeodactylum tricornutum* to lift over the gene models from Phatr3, a previously annotated reference genome. We used the lifted genes annotation and newly published transposable elements to map the epigenome landscape, namely DNA methylation and post-translational modifications of histones. This provides the community with PhaeoEpiView, a browser that allows the visualization of epigenome data and transcripts on an updated and contiguous reference genome, to better understand the biological significance of the mapped data. We updated previously published histone marks with a more accurate peak calling using mono instead of poly(clonal) antibodies and deeper sequencing. PhaeoEpiView (https://PhaeoEpiView.univ-nantes.fr) will be continuously updated with the newly published epigenomic data, making it the largest and richest epigenome browser of any stramenopile. In the upcoming era of molecular environmental studies, where epigenetics plays a significant role, we anticipate that PhaeoEpiView will become a widely used tool.

## Introduction

Diatoms are one of the most abundant and highly diverse microbial eukaryotes, contributing to 20–25% of the Earth’s global carbon dioxide fixation and approximately 40% of marine primary production^1–3^. Diatoms are highly successful and ubiquitous, occupying large territories including marine, freshwater, sea ice, snow and even moist terrestrial habitats.

The marine diatom *P. tricornutum* is one of the most comprehensive and attractive models in microalgae. It is commonly used as a model organism for answering fundamental questions about diatoms biology and ecology. This species is widely used in biotech applications as it was proven to be a source of various high value compounds such as eicosapentaenoic acid and fucoxanthin^4,5^, a bioremediating agent for its capacity of growth in toxic environments^6^, a cell factory for producing several recombinant proteins of interest and is a commercially viable species for large scale cultivation^7^. Furthermore, *P. tricornutum* is emerging as a unique marine microalga for epigenetic studies, showing interesting patterns of its chromatin landscape with features from animal and plant kingdoms reflecting its evolutionary history^8–12^. Besides gaining fundamental insights into transcriptional regulation and metabolic pathways of microalgae genomes, epigenetics offers the potential to facilitate microalgae breeding programs for the production of biomass and high value substances^13^. While rapid growth rate and metabolic plasticity (photoautotrophy/mixotrophy), ease of transformation and cryogenic preservation are distinguishing features for a model microalga, *P. tricornutum* has become a prominent model organism, due to its sequenced genome and high-quality annotation. This has provided a valuable resource for expanding its molecular toolbox, including expression databases, genetic editing tools and epigenome landscape.

The genome of the model diatom *Phaeodactylum tricornutum* CCAP 1055/1 and the corresponding Phatr2 annotation were published in 2008 using whole genome shotgun paired-end Sanger sequencing (NCBI assembly ASM15095v2)^14^. Subsequently, Phatr3 annotation updated and expanded the gene repertoire to introduce over a thousand novel genes, and performed a comprehensive de novo annotation of repetitive elements uncovering novel classes of transposable elements. This was accomplished by analyzing a combination of 90 RNA-Seq datasets, along with published expressed sequence tags and protein sequences^15^. The first assembly of the genome contained 33 scaffolds among which 12 telomere-to-telomere chromosomes. Using long-read sequencing, Filloramo et al.^16^ re-examined *P. tricornutum* assembly which led to additional sequence information, but did not improve the continuity and chromosome-level scaffolds compared to the original reference genome^16^. An approach combining long reads from the Oxford Nanopore minION platform and short highly accurate reads from the Illumina NextSeq platform, was recently used to reassemble the genome of *P. tricornutum.* This resulted in a significant improvement with the identification of 25 nuclear chromosomes^17^. However, despite this update, the Phatr3 annotation of *P. tricornutum* has not been revised to reflect the new assembly, which is a common occurrence for many species where annotations lag behind the latest improvements in genome assemblies.

While whole-genome sequencing is critical for gaining a deeper understanding of the ecological success of diatoms, DNA sequence is only the basis for understanding how to read genetic programs. Epigenetics which constitutes an additional layer of inheritable information, superimposed on the DNA sequence is also critical in this process. It has already been proposed that the ecological success of phytoplankton is also due to the adaptive dynamics conferred by epigenetic regulation mechanisms, because point mutation-based processes may be too slow to permit adaptation to a dynamic ocean environment^18^. The epigenetic changes may lead to chromatin modifications, which may cause a stable alteration in transcriptional activity even after withdrawal of the triggering stress^19^. Pioneering work drew a comprehensive map of epigenetic marks, including several permissive and repressive post-translational modifications of histones (PTMs) and DNA methylation in *P. tricornutum*, and showed their contribution to mediating the response of diatom cells to environmental factors^8,9,12^.

**Figure 1:**
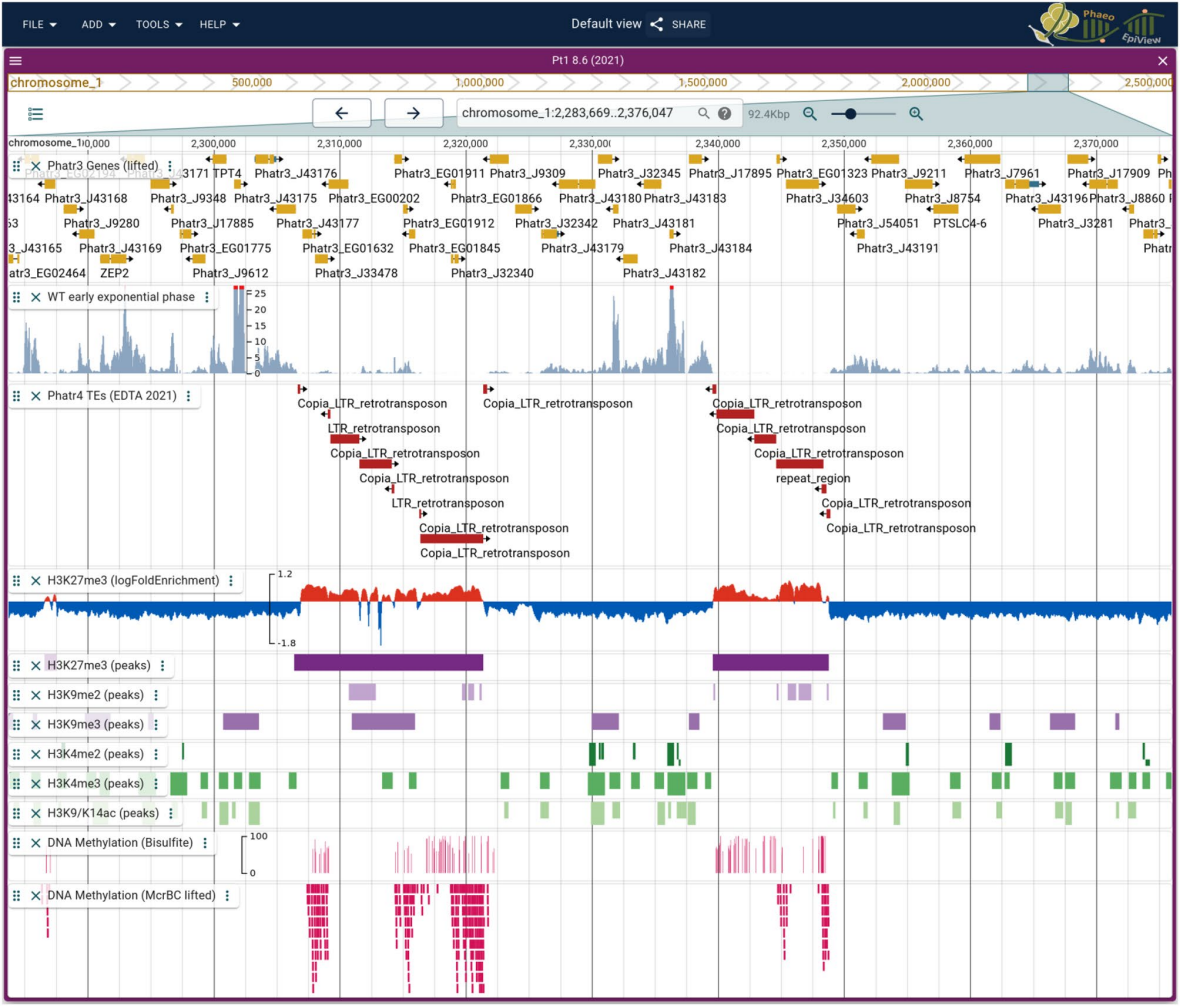
Snapshot of PhaeoEpiView browser illustrating the different tracks of genes, transposable elements, histone marks and DNA methylation. Both peaks and log2 fold enrichment between IP and Input are displayed for H3K27me3.

An important molecular toolbox is available in *P. tricornutum* including epigenomic data which are only found in the partial assembly. To make such a resource available on the newly assembled genome, we used the 25 to 25 telomere assembly to map the epigenetic data namely PTMs and DNA methylation that were previously published^8,9,20^. We revisited two histone marks to improve the mapping and performed chromatin immunoprecipitation using monoclonal instead of polyclonal antibodies reported in our previous study. Prior to this, we lifted the Phatr3 annotation using a gene based approach and integrated the various tracks onto a browser called PhaeoEpiView (Fig. 1).

## Materials and methods

### Culture and growth conditions

*Phaeodactylum tricornutum* Bohlin Clone Pt1 8.6 (CCMP2561) cells were obtained from the culture collection of the Provasoli-Guillard National Center for Culture of Marine Phytoplankton (Bigelow Laboratory for Ocean Sciences, USA). Constantly shaken (100 rpm) cultures were grown at 19 °C, 60 µmol photons/m^2^/s and with a 12 h light/12 h dark photoperiod in sterile Enhanced Artificial Sea Water (EASW) medium^21^. For Chromatin immunoprecipitation-sequencing, cultures were seeded at 50.000 cells/ml in duplicates and grown in 1000 ml Erlenmeyer flasks until early-exponential at 10^6^ cells/ml. Culture growth was measured using a hematocytometer (Fisher Scientific, Pittsburgh, PA, USA).

### Liftoff analysis

Using minimap2 and Liftoff tools, exons are aligned first to preserve the gene structure of the Phatr3 annotation^22,23^. Minimap is used with 50 secondary mappings, end bonus of 5 and chaining score of 0.5. Genes are lifted and considered mapped successfully if the alignment coverage and sequence identity in the child features (usually exons/CDS) is ≥ 50%. We used two different strategies of annotation of gene structure on the new assembly: 1. Whole genome-based reference alignment of all genes. 2. Chromosome-by-chromosome liftover.

RNA sequencing from the reference ecotype (Pt1 8.6) was then aligned to both the current and previous genome versions with BWA-mem2 2.2.1^24^ and transcripts quantification was performed with HTSeq 0.13.5^25^ using the gene annotation corresponding to each genome. Transcript quantification and gene lengths were then compared between the two versions using R (URL https://www.R-project.org/).

### Chromatin extraction and immunoprecipitation

Chromatin isolation was performed as described previously^26^ with few modifications. Briefly, the incubation step in buffer II is repeated several times until the pellet becomes white. Each ChIP-Seq experiment was conducted in two independent biological replicates. Monoclonal antibodies from Cell Signaling Technology were used for immunoprecipitation, H3K9me3 (13969), and H3K27me3 (9733). Satisfactory monoclonal antibodies were not accessible for the other histone marks examined in previous studies.

### ChIP-Seq analysis

Pair-end sequencing of H3K9me3, H3K27me3 ChIP and input samples was performed on NovaSeq 6000 with read length of 2 × 150 bp. Previously published ChIP sequencing for H3K9me2, H3K9me3, H3K4me2, H3K27me3, H3K9/K14Ac and H3K4me3 were retrieved from NCBI’s Gene Expression Omnibus accessions GSE68513 and GSE139676^9,10^. Raw reads were filtered and low-quality read pairs were discarded using Trim Galore 0.6.7 (https://doi.org/10.5281/zenodo.5127899) with a read quality (Phred score) cutoff of 20 and a stringency value of 3 bp. Using the 25 to 25 telomere assembly published in 2021 as a reference genome, the filtered reads were mapped using Bowtie2 2.4.5^27^. We then performed the processing and filtering of the alignments using Samtools 1.15 “fixmate -m” and “markdup-r” modules^28^. Two biological replicates for each ChIP were performed and read counts showed a good Pearson correlation by Deeptools multiBamSummary v3.5.1 with a bin size of 1000 bp^29^.

To identify regions that were significantly enriched, we used MACS2 v2.2.7.1^30^ on the combination of the two replicates with “callpeak --qvalue 0.05 --nomodel –SPMR --bdg” options. In addition, extension size was set to the arithmetic mean of the two IP replicates fragment size for each mark, as determined by MACS2 predicted module with “-m 2 70” MFOLD value. Furthermore, “--broad” calling mode was activated for H3K9me2 that was previously described as broad histone marks. For the narrow marks H3K4me2, H3K9/K14Ac and H3K4me3, peaks summits were called with “--call-summits”. Following previously published work, SICER2 v1.0.3^9^ was used with “-w 200 -g 600 -fdr 0.05” to call peaks for H3K27me3. For H3K9me3, we used two different peak detection methods SICER and MACS2 simultaneously to call peaks. We only kept peaks called by MACS2 that had an overlap with a peak called by SICER2. Output normalized Fold Enrichment signal files were generated with MACS2 v2.2.7.1 “bdgcmp” module and transformed to BigWig using Deeptools bedGraphToBigWig. Then, Pearson correlation between our new data and previously published data for H3K9me3 and H3K27me3 was performed using Deeptools plotCorrelation.

### ChIP-qPCR

Chromatin Immunoprecipitation (ChIP) was conducted as described previously^26^. DNA concentrations were quantified with the Qubit dsDNA BR Assay Kit (Thermo Fisher Scientific). Quantitative PCR experiments were performed using LightCycler DNA Master SYBR Green Mix on the Biorad LightCycler. IP enrichments were assayed with the following protocol: 1 μl of immunoprecipitated DNA samples (IP), input and mock DNA were mixed with 5 μl LightCyclerW DNA Master SYBR Green I 2X, 3 μl forward/reverse primers (1 μM), and 1 μl H2O. For each IP sample, the enrichment of certain histone modification on specific loci was calculated with the following equation: %Enrichment level = 100/2^(Cq[ChIP] − (Cq[Input] − Log2(Input Dilution Factor))^. We performed ChIP-qPCR validation using monoclonal antibodies against H3K9me3 and H3K27me3. Primers designed on randomly selected genes and used for ChIP-qPCR validation were listed in Table S1.

### Expression analysis

Early and late exponential growth phase Illumina RNA-seq data (SRR5274697, SRR5274696, SRR5274695 and SRR5274694) from Ref.^31^ were trimmed using Trim Galore 0.6.7 with a read quality (Phred score) cutoff of 20 and a stringency value of 3 bp. Technical replicates were merged and mapped to the reference assembly with STAR 2.7.10a^32^. Primary alignments only were processed with Deeptools bamCoverage 3.5.0 with “--normalizeUsing BPM --ignoreDuplicates --centerReads” to generate normalized coverage files to be displayed in PhaeoEpiView.

### DNA methylation analysis

McrBC DNA methylation annotation data from Ref.^8^ was lifted from the previous assembly to the new 25 chromosomes using Liftoff. Bisulfite sequencing data^20^ were processed with Bismark v0.22.3^33^ and methylated regions having less than 50% methylated reads or less than 5 supporting reads were filtered out. Nanopore DNA methylation data from Ref.^17^ was retrieved, converted to a compressed quantitative data format (BigWig) and added to the Jbrowse instance. Pairwise Pearson correlation coefficients of the three DNA methylation data tracks were then calculated and plotted as a heatmap with multiBigwigSummary and plotCorrelation tools from deepTools package v3.5.1^29^, using 10 kbp bins size.

## Results and discussion

Despite being an established model, the genome and annotation of *P. tricornutum* are not concordant and the existing epigenetic resources generated so far lack a well-defined framework for accurate and user friendly utilization. To address this limitation, we sought to establish a coherent resource rendering the multiple genomic and epigenomic sequencing data sets exploitable from a single platform which we named PhaeoEpiView.

Because *P. tricornutum* recent genome assembly and annotation exhibit inconsistencies, PhaeoEpiView was built using two steps (i) Phatr3 gene annotation lifting onto the new 25 chromosomes assembly (GCA_914521175.1) and (ii) mapping of the previously published epigenetic marks and transcripts on the new assembly. Next, we proceeded with Phatr3 gene annotation lifting onto the new 25 chromosomes assembly (Phatr4). In the first step, instead of whole genome-based comparison, we adapted a gene-based sequence alignment for lifting the annotation from Phatr3 to Phatr4 assembly. Features such as mRNA, CDS and exons from the reference Phatr3 were used to infer genes and transcripts in target assembly. Unplaced genes and genes with extra copy number are tagged and separated (Table S2). Any remapped duplicated genes are also included in Table S2 for thoroughness. Out of the 12,178 genes from Phatr3 annotation, 11,739 were lifted successfully (Supplementary File 1). The new assembly is missing around 439 genes that were not mapped and lifted from the reference Phatr3 genome. But remapping of these 439 unaligned genes resulted in recovery of 295 genes. These are duplicated genes in the reference annotation that are assembled into one copy in the newly assembled genome. This 1.8% (144 genes) that are not found in the telomere-to-telomere (T2T) assembly are assembled into a unique set of genes. These non-transferable genes might be part of retrotransposons or repetitive element regions. We could recover these genes by allowing overlapped gene model remapping. This suggests that the telomere-to-telomere genome assembly represents a comprehensive and thorough assembly, and that our liftover gene annotation successfully captures all important functional genes.

In order to validate the lifted annotation, we aligned RNAseq reads to both the previous and the new genome version, then compared every gene quantification. Out of 8754 genes with a non-zero transcripts count, 5795 had a difference of quantification (Fig. S1) and 5836, a difference of length lower than ± 0.1% between Phatr3 and Phatr3_lift (Phatr4). Moreover, 7687 of these genes had a difference of quantification and 8609 of length below ± 5%. Missing genes were then examined: 178 out of 439 (40%) were found to be located on short regions that are no longer present in Phatr4 assembly according to whole-genome alignment provided in Giguere et al.^17^, half of them clustered on previous chr_5 and chr_21 (Table S2). Most of the remaining 261 missing genes showed similarity to already lifted genes suggesting that they are either duplicated or allelic. Both scenarios suggest the presence of haplotigs in the new assembly that are now collapsed leading to previously falsely duplicated genes competing for the same genomic location on the T2T assembly. Consequently, this led to incorrect interpretation of duplicated genes or genomic regions as two distinct copies generating artificial duplications in downstream analysis.

In the second step, transposable elements annotation available from Giguere et al.^17^ was added to PhaeoEpiView as Phatr4 TEs track. Finally, previously published expression data at two different time points, DNA methylation and PTMs tracks were implemented in the browser, and systematic comparison was made with the previous assembly mapping using unchanged regions as anchors (Supplementary File 1). Our DNA methylation data obtained through bisulfite conversion and MCrBC digest was compared to DNA methylation data from Nanopore sequencing. The results demonstrated an overall good coefficient correlation indicating consistency between the three data sets (Fig. S2).

**Figure 2:**
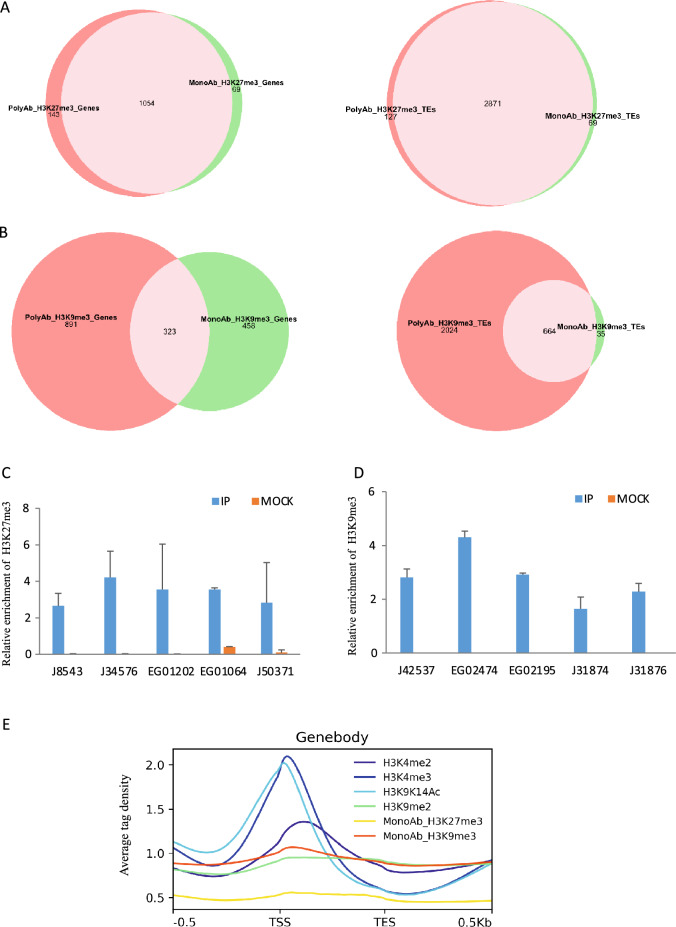
(**A**) Venn diagram of H3K27me3 on genes (left) and TEs (right). (**B**) Venn diagram of H3K9me3 on genes (left) and TEs (right). (**C**) ChIP-qPCR validation of randomly chosen loci for H3K27me3 (**C**) and H3K9me3 (**D**). Mock values are indistinguishable from 0 in (**C**). (**E**) The enrichment profile of H3K4me2, H3K4me3, H3K9_14Ac, H3K9me2, H3K9me3MonoAb, H3K27me3MonoAb along genes (upstream TSS, coding region, downstream TES). Average tag density is the number of sequence reads per gene.

For more accuracy and homogeneity of the used data, chromatin immunoprecipitation with deep sequencing was carried out using commercially available monoclonal antibodies to replace two marks, H3K27me3 and H3K9me3 for which polyclonal antibodies were used in the previous study^9^ (Fig. 2A–D, Fig. S3A,B). With few exceptions, H3K27me3 antibody used in this study provided an overall similar pattern of distribution over the genome supported by a strong Pearson’s correlation coefficient (0.80) (Fig. S3A). We used ChIP-qPCR and validated randomly chosen loci which further supports the accuracy of H3K27me3 mapping (Fig. 2C). H3K27me3 polyclonal antibody was previously tested in a peptide competition assay and showed very little cross reactivity which is in line with the strong correlation coefficient between the two mapped datasets (^26^, this study). However, 69 genes and 127 TEs were not recovered using H3K27me3 monoclonal antibody indicating that these regions were false positives in the previous mapping using the polyclonal antibody (Fig. 2A).

Using a monoclonal H3K9me3 antibody, the current study revealed a negative correlation with the previous work that employed a polyclonal antibody, demonstrating a higher specificity of the former (Fig. S2D). The polyclonal antibody which was not tested for cross reactivity is likely recognizing other epitopes/marks such as H3K9me1/me2. Randomly chosen loci, targets of H3K9me3 monoclonal antibody were validated by ChIP-qPCR indicating the reliability of chromatin immunoprecipitation and mapping (Fig. 2D).

Using monoclonal H3K9me3 antibody revealed a different pattern of distribution of genes and transposable elements. H3K9me3 monoAb covered fewer genes and transposable elements which is expected considering the specificity of the antibody. H3K9me3 targeted mainly genes compared to the previous mapping where the majority of targets were TEs. A total of 323 genes and 664 TEs were shared between the two antibodies (Fig. 2B). Both H3K9me3 and H3K37me3 are broad marks and do not have clearly defined peak summits compared to more localized marks such as lysine9/14 acetylation of histone H3 (Fig. 2E). For both marks, peaks overlapped significantly downstream transcriptional start sites (TSS) over gene bodies (Fig. S3E,F).

**Figure 3:**
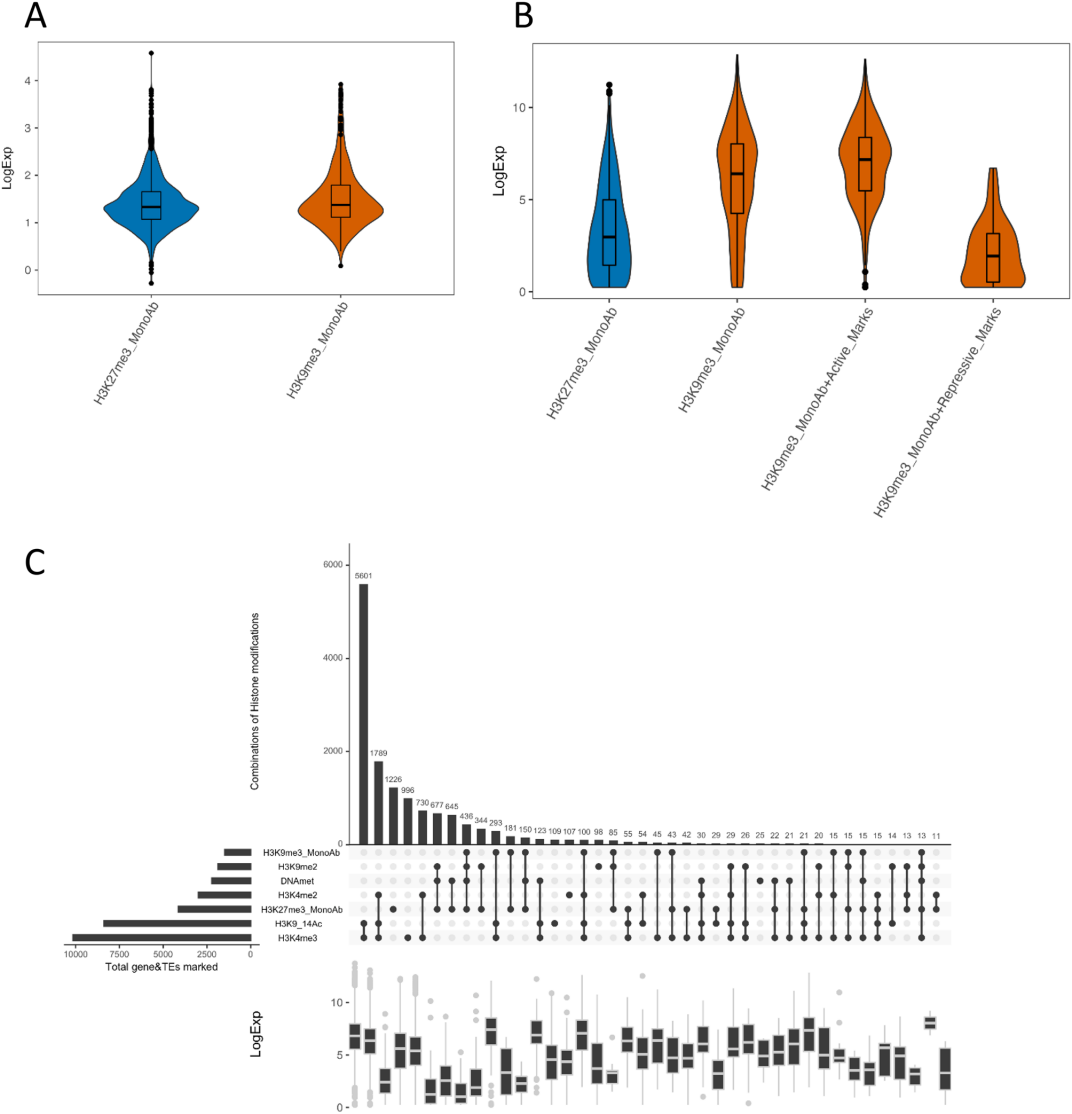
Expression profiles of genes and TEs marked by different histones marks in *P. tricornutum*. Expression levels of TEs (**A**) and genes (**B**) marked by H3K9me3 or H3K27me3 alone or combined with repressive or permissive marks. (**C**) Co-occurrence analysis of epigenetic marks in *P. tricornutum*. A co-occurrence plot for the ChIP-seq peaks of different histone modifications. For each set of intersection, a black filled circle is drawn. The size of the intersections is depicted in vertical bar. The vertical black line connects the different dataset that intersect. The number of genes and TEs for each chromatin state is shown on the top of the bars.

Considering the differences in targeted genic regions using H3K9me3 monoclonal antibody compared to previous mapping, we looked at the effect of H3K9me3 localization on transcriptional regulation of genes and TEs. First, we considered H3K9me3 only marked genes and TEs, and found a clear repression when TEs are marked by H3K9me3 with levels comparable to H3K27me3 marked TEs, while genes seem to show moderate downregulation compared to marks known to be repressive such as H3k27me3 (Fig. 3A,B). When we consider H3K9me3 co-occurrence with other histone marks, there is a downregulation of genes when it co-occurs with repressive marks and an upregulation when co-localized with permissive marks (Fig. 3C). Overall, H3K9me3 seems to be a repressive mark in *P. tricornutum*.

PhaeoEpiView was implemented as a Jbrowse2 instance^34^ and made public on a virtual machine hosted at Nantes University datacenter (Fig. 1). It can currently display one track for each of the genes, TEs, transcript levels, McrBC, Bisulfite-seq and Nanopore DNA methylation tracks and five histone PTMs (H3K9/14Ac, H3K4me2, H3K4me3, H3K9me2, H3K9me3, H3K27me3). The browser will be regularly updated with relevant epigenomic data when published in the future, turning PhaeoEpiView into a dynamic platform for a comprehensive genomic and epigenomic resource of the model microalgae *P. tricornutum*.

## Conclusion

PhaeoEpiView is an open source browser that provides an up-to-date genome and epigenome view of the model diatom *Phaeodactylum tricornutum*. With the lifted genes annotation, the epigenome and transcriptome landscapes can be visualized on a fully assembled genome providing an accurate view of epigenetic regulation of genes and TEs which was incomplete on the previously fragmented genome. In addition, PhaeoEpiView allows users to upload their own tracks in private sessions for visualization and data interpretation purposes. PhaeoEpiView is intuitive, user-friendly and stands out as the first epigenome browser for a photosynthetic unicellular species. PhaeoEpiView will undoubtedly contribute to boosting fundamental and applied research in microalgae and other single celled species.

## Supplementary Information

Supplementary Legends.

Supplementary Figure S1.

Supplementary Figure S2.

Supplementary Figure S3.

Supplementary Table S1.

Supplementary Table S2.

Supplementary Table S3.

## Data Availability

The datasets generated and analysed during the current study are available in the BioProject repository as PRJNA911955 (Biosamples SAMN32216962, SAMN32216963, SAMN32216964, SAMN32216965, SAMN32216966, SAMN32216967, SAMN32216968, SAMN32216969).
