# Supplementary material for: PhaeoEpiView: an epigenome browser of the newly assembled genome of the model diatom Phaeodactylum tricornutum
Source: Sci Rep. 2023 May 23;13:8320. doi: 10.1038/s41598-023-35403-1 (PMC10206091; doi:10.1038/s41598-023-35403-1)
Supplement: Supplementary file 1 — Supplementary Legends. [file 41598_2023_35403_MOESM1_ESM.docx]

**Supplementary Figure 1**. Comparison of RNA-seq quantification per gene on the 2008 (33 scaffold/chromosomes) and 2021 (25 chromosomes) assembled genomes. Two RNA-seq replicates were used.

**Supplementary Figure 2**. Heatmap of Pearson correlation coefficients between the 3 DNA methylation tracks available on PhaeoEpiView: McrBC, Bisulfite and Nanopore base calling. Pearson correlation values are displayed in each square.

**Supplementary Figure 3**. Pearson coefficient correlation of replicates for H3K27me3 monoAb (A), H3K9me3 monoAb (B), mono and polyAb of H3K37me3 (C), mono and polyAb of H3K9me3 (D). (E) (F) ComputeMatrix, plotProfile and plotHeatmap were used to summarize and cluster multiple bigwig scores over genomic intervals and show the resulting k-means clustering of ChIP-seq signals of both H3K9me3 and H3K27me3 around the transcription start site of genes. The top panel shows the average signal of H3K9me3 and H3K27me3 monoclonal and different cluster of regions (Gene and TE). The bottom panel shows summary profiles where colors correspond to the observed frequency of the signal within each cluster.

**Supplementary Table 1.** List of primers used in qPCR validation study.

**Supplementary Table 2**. A comprehensive list detailing the genes that were not successfully recovered through the lifted annotation process, with their respective coordinates. Any remapped duplicated genes are also included in the list.

**Supplementary File 1.** GFF3 file annotating *Phaeodactylum tricornutum* 2021 assembly with Phatr3 lifted genes.
