# Supplementary figures and images for: PhaeoEpiView: an epigenome browser of the newly assembled genome of the model diatom Phaeodactylum tricornutum
Source: Sci Rep. 2023 May 23;13:8320. doi: 10.1038/s41598-023-35403-1 (PMC10206091; doi:10.1038/s41598-023-35403-1)

**Replicate 1 - 8754 values**

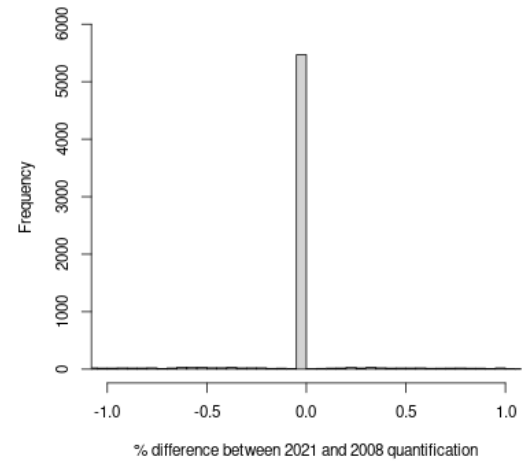

**Replicate 1**

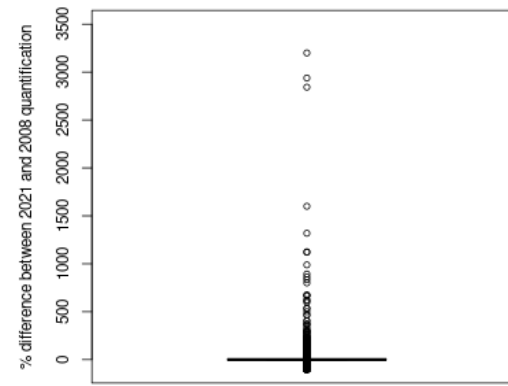

**Replicate 2 - 8754 values**

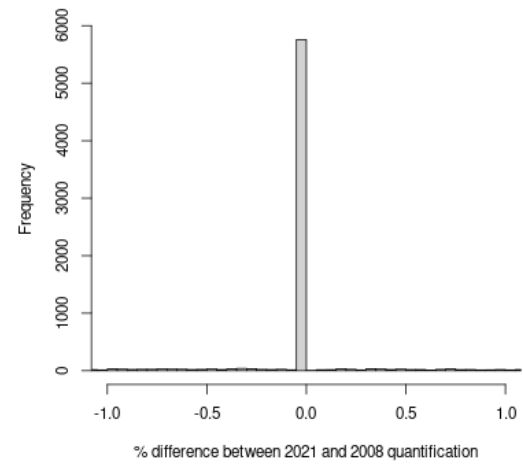

**Replicate 2**

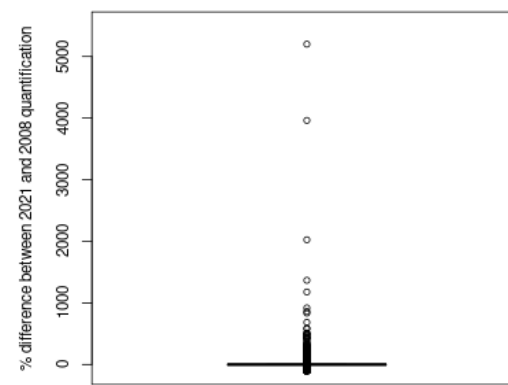

Supplement: Supplementary file 2 — Supplementary Figure S1. [file 41598_2023_35403_MOESM2_ESM.pdf]

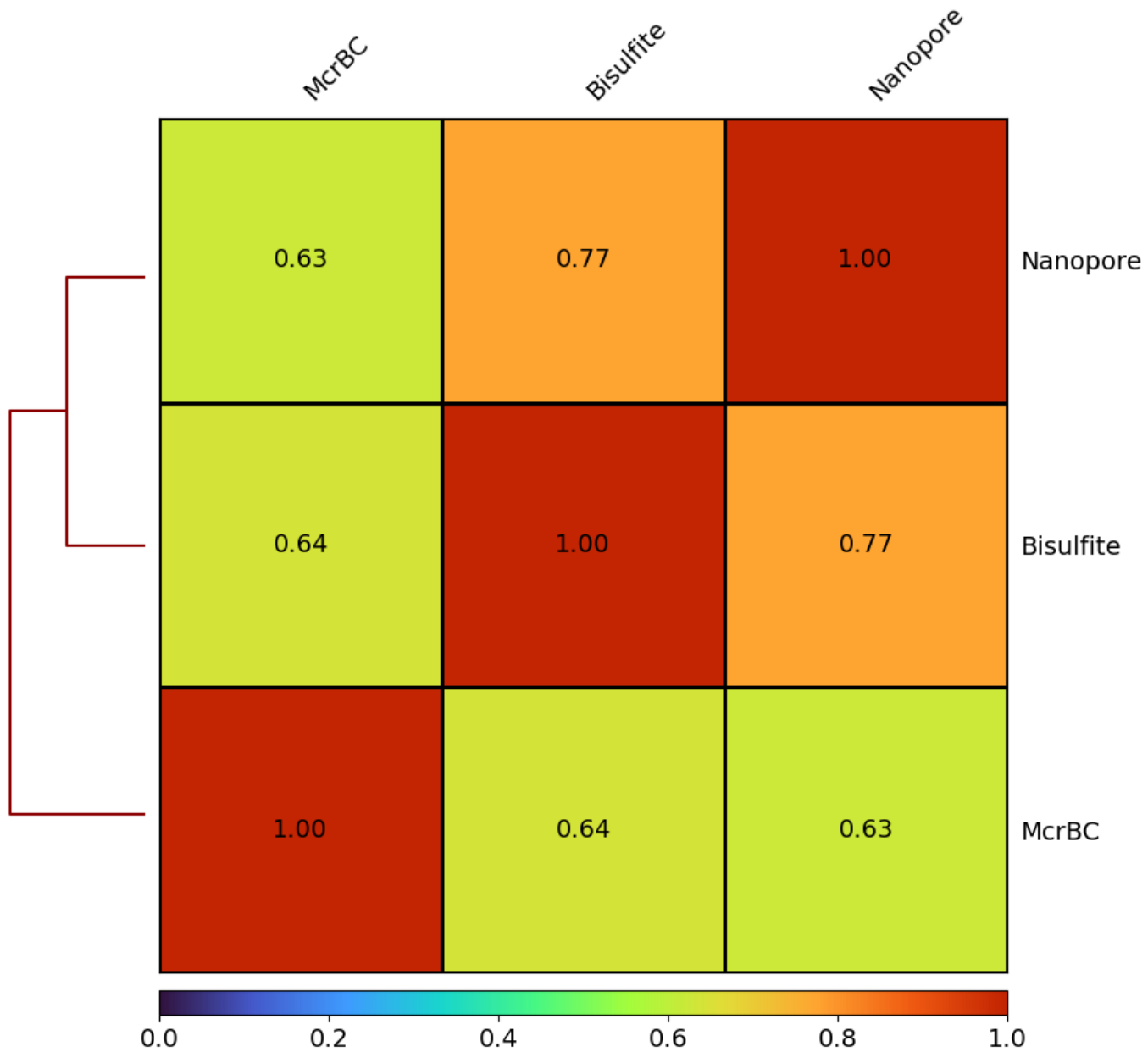

Supplement: Supplementary file 3 — Supplementary Figure S2. [file 41598_2023_35403_MOESM3_ESM.pdf]

## Slide 1
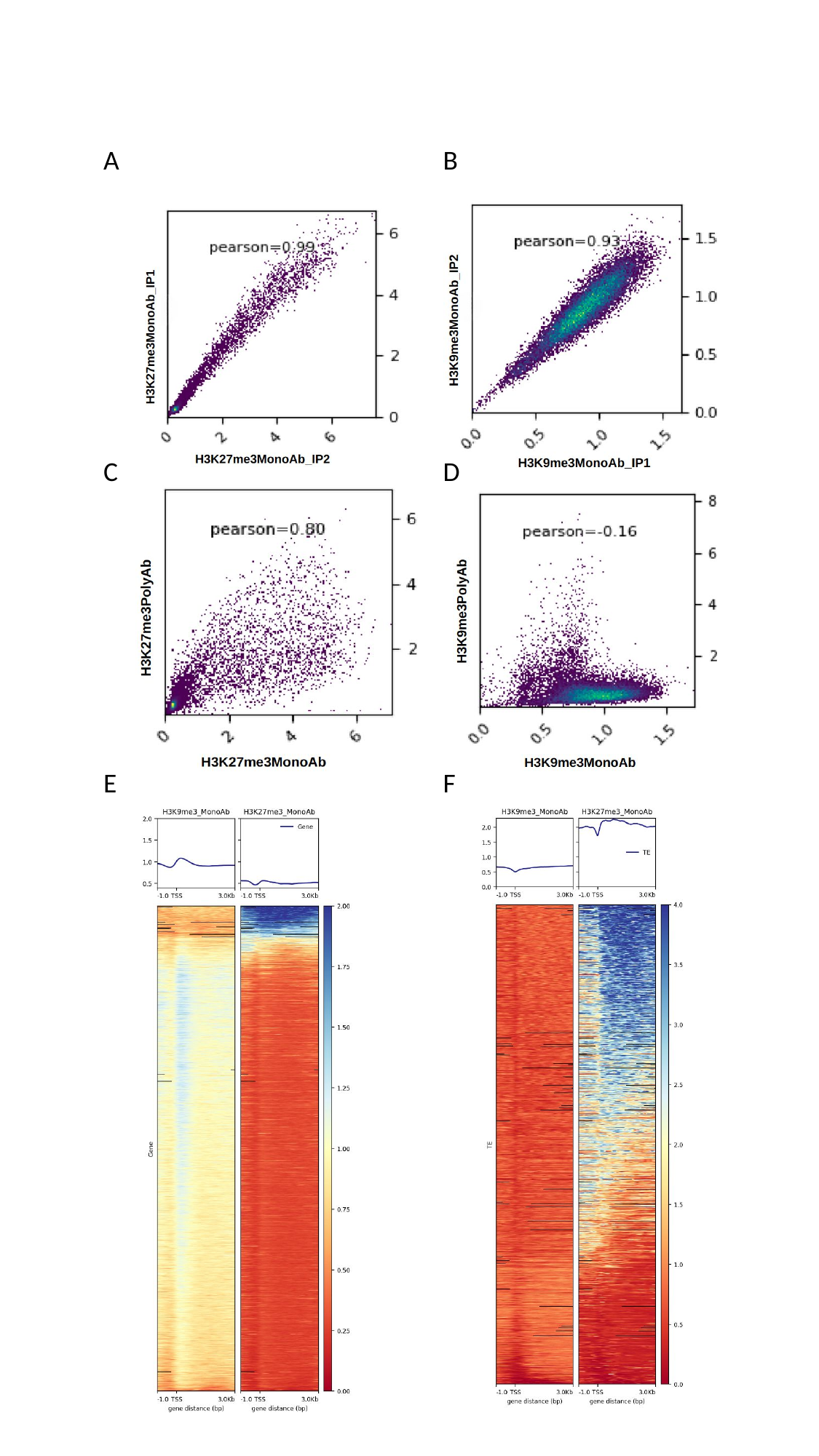

A
B
C
D
E
F

Supplement: Supplementary file 4 — Supplementary Figure S3. [file 41598_2023_35403_MOESM4_ESM.pptx]
