# Supplementary material for: PhaeoEpiView: an epigenome browser of the newly assembled genome of the model diatom Phaeodactylum tricornutum
Source: Sci Rep. 2023 May 23;13:8320. doi: 10.1038/s41598-023-35403-1 (PMC10206091; doi:10.1038/s41598-023-35403-1)
Supplement: Supplementary file 5 — Supplementary Table S1. [file 41598_2023_35403_MOESM5_ESM.pdf]

Supplementary table 1. Primer sequences used for qPCR analysis

Primer name*	Sequence
1 EG02195 Fwd	TCGTTATTTGTTGGATTTGCACA
2 EG02195 Rev	GGATAGGATGATTGGGCTCGA
3 J42537 Fwd	TCCTCTATCGTTCCAGCGTG
4 J42537 Rev	ACAGTTGATAGGTTTAGGGCCA
5 J31876 Fwd	CGCAAAAGCGTTATATGGCC
6 J31876 Rev	CCACATATCAGGGTCCGCTT
7 EG02474 Fwd	GCCGTCACCATAACTCTCGA
8 EG02474 Rev	TGAAGGAAGCGGCTTGGTAG
9 J31874 Fwd	CGCCCTCGGTAACAAAATCA
10 J31874 Rev	GTGTCTGTTACGGGTGTTGC
11 J8543 Fwd	GCGGTGGAGATCTCGGTATA
12 J8543 Rev	GTCAGCCAGCGAATAGAACG
13 J34576 Fwd	ACAGCAATACCCCACACCGG
14 J34576 Rev	AGCAGCGGTTCCATCGACTT
15 EG01202 Fwd	ATGAAGAGCCAGGATGGATG
16 EG01202 Rev	ACATTTGTATTGCCGCTTCC
17 EG01064 Fwd	GCTATCACTACATTGGCCCAC
18 EG01064 Rev	CGCTCACATCCATCCACGTT
19 J50371 Fwd	GGGACACGACTCGAAACGGA
20 J50371 Rev	CCGTTGTTCTTGTCGTGGTGG

*Primers from 1 to 10 were used for validation of H3K27me3 marked loci. From 11 to 20 were used for validation of H3K9me3 marked loci
